# A Clinical Lecture

**Published:** 1886-02

**Authors:** W. S. Elkin

**Affiliations:** Atlanta, Ga.; Lecturer on Venereal Diseases in the Southern Medical College


### A CLINICAL LECTURE.

BY W. S. ELKIN, M. D., ATLANTA, GA.

LECTURER ON VENEREAL DISEASES IN THE SOUTHERN MEDICAL COLLEGE.

Reported for THE ATLANTA MEDICAL AND SURGICAL JOURNAL.

The first case I have to show to-day, gentlemen, is a young man who presented himself at the hospital during the holidays, when the lectures had closed and most of you were out of the city. At that time he was suffering from phimosis, due to a severe balanitis and a hard indurated mass, which seemed to be between the skin and mucous membrane of the prepuce and covered at least one-third of the left side of the glans penis. The history of the case as elicited from the patient was very imperfect and unsatisfactory. I could only ascertain that the present state of affairs had existed since last September, and that the sub-preputial discharge and inflammation were increasing daily, and for more than a month the patient had not been able to expose the glans penis. The discharge from the extensive balanitis had so excoriated the skin around the preputial orifice that great pain was experienced in passing water. I found the post-cervical glans and the ganglia in both groins enlarged, which caused me to suspect that the induration you see here was of a specific nature. He had used sub-preputial injections in order to allay the inflammation, but to no avail. I concluded to expose the glans penis by the ordinary operation for circumcision, which you have seen me perform several times during the present session upon patients in this hospital. The phimosis forceps could not be used, but with a grooved directory and curved bistoury, I succeeded in dividing the prepuce in the median line as far back as the fossa glandis. This procedure brought to view the ulcerations that were confined chiefly to the mucous membrane of the prepuce and were removed in the ablation of the side flaps. In removing the prepuce on the left side, the indurated mass above referred to was incised and bled freely, in an oozing manner, until the edges of the wound were brought together by a continuous suture of very fine iron-dyed silk. Iodoform was sprinkled over the wound and successive strips of isinglass plaster were wrapped around the wound to exclude the air. The patient never went to bed (although he was directed to do so), but continued his occupation, which was that of an engineer, and yet in the face of this risk he has made a most excellent recovery. The wound, as you see, has healed by first intention. Even the induration which was incised throughout its whole length is as nicely united as, the healthy prepuce of the opposite side. It is true we have a good result here, but an operation of this kind should be avoided or deferred when the phimosis is due to an acute inflammation, which generally can be subdued by rest and proper treatment. At the time of this operation, two weeks ago, I suspected the true nature of this induration, yet no secondary manifestations had appeared to give me positive proof of the correctness of my diagnosis. To-day we find a characteristic erythematous eruption scattered over the entire trunk, arms and legs of this man, which leaves no doubt as to the specific character of the disease. This is one of the first eruptions to make its appearance, and generally occurs from six to twelve weeks after the initial lesion, and is characterized by rose-colored spots, not elevated above the level of the skin, and is known as erythema maculatum. There is a general febrile condition of the patient, accompanied with headache and a feeling of malaise that precedes for a few days the appearance of this eruption. We have here a case of syphilis to deal with, and our treatment shall be directed to that end. I will order for this man one-fourth of a grain of proto-iodide of mercury, to be taken three times a day after meals; to be increased if necessary, as the case demands. This patient will appear from time to time at subsequent clinics, when we shall be able to see the result of our treatment.

The next case I wish to present is a negro woman twenty-five years of age, who is suffering from syphilis, but in a more advanced stage than the man that was just before you. Observe her general appearance. She is very much emaciated, and the skin from the soles of her feet to the collar around her neck is thickly covered with a pustular eruption. Again, in this case, as in the other, the history is sadly unsatisfactory. She cannot tell when she contracted this disease, or with any certainty how long she has had these and other eruptions on her body. You will remember that the rose-colored blotches that were so manifest on the body of the young man were smooth, superficial, and not in the least elevated above the surrounding skin, and in the course of time this erythema gradually fades away and finally disappears with only a slight desquamation of the cuticle. After the disappearance of this macular eruption, sometimes even before it has entirely vanished, there comes on another variety of this eruption, known as the erythema papulatum, which is somewhat elevated above the level of the skin, is more or less scaly and seems to be the intermediate link between the erythematous and papular syphilides. Following the erythema papulatum comes the papular syphilides, which are characterized by two forms, the small and the large papular syphiloderms, which are sometimes called respectively the miliary and lenticular syphilides. The former occurs upon the trunk, face, arms and legs as small pin-head sized, red-pointed elevations, which have their apices sometimes covered with scales. They soon lose this red hue, becoming purple in color and finally disappear, leaving no trace behind. With the exception of being more thickly and widely disseminated, they resemble the acne that is so often seen on the face and back, and for this reason is sometimes known as acne syphilitica. Often before this eruption has disappeared the lenticular form of this same variety makes its appearance. It differs from the miliary in size, being broad, flat papules, varying in size from a split pea to a ten cent piece, more elevated above the surrounding tissue, and having the summit of the papules crowned with thicker and darker scales. The papules are of a dull red color and are not so widely diffused as the erythema and the miliary papules, occurring generally upon the palms of the hands, soles of the feet, between the fingers and toes, on the scalp at the margin of the hair, about the genitals of both sexes, at the angle of the mouth, on the thighs and around the anus. When this eruption occurs upon the palms of the hands, the papules sometimes coalesce, the scales that crown the apices peel off and the lesions become fissued and bleed, and by the admixture of blood and scales we have a condition of affairs that is difficult to distinguish from eczema of the palms. But if you will remember this latter affection is rare and that papular syphilides of the palms are by no means uncommon, you will not often be misled in making your diagnosis. In eczema of the palms we have burning heat and itching, which is not present in specific eruptions, and of course, if you can always get the history of syphilis you can at once diagnose the disease. It is safe to say that the vast major-ity of cases in which we find a squamous eruption of the palms of the hands may be regarded as syphilis.

I am sorry that we have no case here to-day to illustrate the papular variety of syphilitic eruptions. I have spoken of it, how-ever, that you might know the gradual change that takes place between erythema that I have shown you and the pustular form that you see covering this woman's body. The secondary mani-festations may come in succession in regular order, with intervals between them, in which the skin is entirely free from any eruption, or they may come out with a rush one upon the other when we may find macules, papules and pustules all present at the same time. Besides the pustules, in this case, which are thickly scattered overthe body, we find the inguinal, epitrochlear and post-cervical glans still enlarged, presenting what is known as syphil-itic adinitis. The tonsils and fauces are more or less congested, and by careful observation we are able to detect two mucous patches, one on the buccal mucous membrane of the left side, and the other just beneath the end of the tongue. There are other symptoms that should always be looked for, and if present confirm the specific character of the disease. This eruption is deeper seated than the papular, beginning in the true skin instead of the epidermis, and pushing itself above the level of the skin and presenting small pointed elevations that are crowned with pustules. The pustules increase until they may reach the size of a split-pea or finger-nail, and may occupy the whole base upon which they are seated. They are round, yellow in color, and generally distended with pus. These pustules are not umbilicated as are the eruptions in variola, and if they progress favorably, dry up and leave behind flakes of dry epidermis with a discoloration of skin beneath, but if any of them are broken, the pus that exudes forms a crusty scale which if removed reveals a slight ulceration. The pustules are thickly scattered over the legs, arms, and trunk not differing in this respect from the erythematous and papular syphilides. I hope soon to have an opportunity to speak to you concerning the tertiary forms of syphilis as well as the syphilides of the mucous membrane and special organs. We will give this woman a mixed treatment of mercury and iodide of potash. I shall prescribe the following:

℞ Hydrag. biniodidi........................ gr. ii.

Pot. Iodidi............................ ʒiv.

Tr. gentianæ............................

Aquæ................................. aa ℥ii.

M.—Sig: Teaspoonful well diluted in water three times a day after meals.

It will be our object to get the patient under the influence of this medicine as soon as possible, and to do this it may be necessary to increase the dose from time to time, watching closely the condition of the gums in order to avoid salivation. Owing to her anæmic condition we will also give her cod liver oil, and endeavor to improve her general health. The constitutional treatment should be be kept up for at least two years.

